# AI and narrative embeddings detect PTSD following childbirth via birth stories

**DOI:** 10.1038/s41598-024-54242-2

**Published:** 2024-04-11

**Authors:** Alon Bartal, Kathleen M. Jagodnik, Sabrina J. Chan, Sharon Dekel

**Affiliations:** 1 The School of Business Administration, Bar-Ilan University, Ramat Gan, 5290002 Israel; 2 Department of Psychiatry, Massachusetts General Hospital, Boston, MA 02114 USA; 3 Department of Psychiatry, Harvard Medical School, Boston, MA 02115 USA

**Keywords:** Birth narratives, Birth trauma, ChatGPT, Childbirth-related post-traumatic stress disorder (CB-PTSD), Maternal mental health, Natural language processing (NLP), Postpartum PTSD, Pre-trained large language model (PLM), Translational research, Preclinical research, Post-traumatic stress disorder

## Abstract

Free-text analysis using machine learning (ML)-based natural language processing (NLP) shows promise for diagnosing psychiatric conditions. Chat Generative Pre-trained Transformer (ChatGPT) has demonstrated preliminary initial feasibility for this purpose; however, whether it can accurately assess mental illness remains to be determined. This study evaluates the effectiveness of ChatGPT and the text-embedding-ada-002 (ADA) model in detecting post-traumatic stress disorder following childbirth (CB-PTSD), a maternal postpartum mental illness affecting millions of women annually, with no standard screening protocol. Using a sample of 1295 women who gave birth in the last six months and were 18+ years old, recruited through hospital announcements, social media, and professional organizations, we explore ChatGPT’s and ADA’s potential to screen for CB-PTSD by analyzing maternal childbirth narratives. The PTSD Checklist for DSM-5 (PCL-5; cutoff 31) was used to assess CB-PTSD. By developing an ML model that utilizes numerical vector representation of the ADA model, we identify CB-PTSD via narrative classification. Our model outperformed (F1 score: 0.81) ChatGPT and six previously published large text-embedding models trained on mental health or clinical domains data, suggesting that the ADA model can be harnessed to identify CB-PTSD. Our modeling approach could be generalized to assess other mental health disorders.

## Introduction

In recent years, artificial intelligence (AI) and related machine learning (ML) analysis strategies have provided promising new options for understanding human language and, in associated applications, improving health care by extracting novel insights from text-based datasets^1–3^. Recent advancements in the field of natural language processing (NLP) computational methods have demonstrated that algorithms can analyze human language and derive understanding similar to human cognition^4^. Pre-trained large language models (PLMs) refer to massive Transformer models trained on extensive datasets^5^. These NLP models have achieved remarkable results in understanding contextual nuances of language in written texts^4^. NLP methods can extract and convert unstructured textual data into structured data, usable for a variety of ML tasks including text classification.

Combined with ML models, language models have been reported as useful in the classification of psychiatric conditions. For example, the MentalBERT and Mental-RoBERTa PLMs were trained to benefit the mental healthcare research community in identifying stress, anxiety, and depression^6^. The mental-xlnet-base-cased^7^ LLM was developed to identify various mental health conditions including stress, depression, and suicide attempts. Both studies found that language representations pretrained in the target domain improve model performance on mental health detection tasks. A survey of NLP models for depression detection showed reasonable accuracy for several models^8^. Language analysis has also been used for detection of schizophrenia with high accuracy^9^.

While these models are often extensively trained on large datasets, in certain circumstances, PLMs can often be used effectively without additional training (zero-shot learning) or training with few examples (few-shot learning)^4^. In zero-shot learning, a model uses its existing knowledge to understand tasks on which it was not explicitly trained^4^. In few-shot learning, a model can make accurate predictions after being trained on a very limited dataset for a particular task^4^.

The Chat Generative Pre-trained Transformer (ChatGPT) large language model (LLM) functions as a conversational agent proficient in following complex instructions and generating high-quality responses in diverse scenarios. Recently, the medical community became intrigued by its capabilities after it demonstrated its proficiency in passing medical board exams^10^, and its potential applications in medical care are wide-ranging^11–13^. In addition to its conversational abilities, ChatGPT has demonstrated remarkable performance on various other NLP tasks, including question-answering^14^, even in zero- or few-shot learning scenarios^4^. In these scenarios, ChatGPT was applied to new tasks with no fine-tuning using no training data (zero-shot learning) or a small number of training examples (few-shot learning)^4^.

In the mental health domain, ChatGPT has been employed for a variety of applications^15–17^, and was able to identify a single patient with schizophrenia and recommend a treatment that aligns with current clinical standards of care^18^. Chat-GPT (GPT-3.5 and GPT-4) has shown significant language processing capabilities in the realm of mental health analysis. For example, the performance of ChatGPT was evaluated in detecting stress, depression, and suicidality, showcasing its strong capability of usefully assessing mental health texts^15^. In another project, ChatGPT was also successful for early depression detection^19^. The performance of ChatGPT in identifying suicide and depression suggests a promising future for PLMs for mental health^15,20,21^.

ChatGPT has presented promising results in the healthcare domain, including applications in healthcare that collect and analyze patients’ clinical information including diagnosis, allergies, and details of previous visits. ChatGPT and similar models have been explored in various tests and clinical deployments for natural language processing in healthcare including applications of therapeutic chatbots, diagnostic assistance, patient education, mental health screening, and clinical documentation. However, in contrast with findings that ChatGPT has shown promising results for healthcare applications, recent reviews^13,22,23^ report that ChatGPT has achieved only moderate performance on a variety of clinical tests. Therefore, careful scrutiny and rigorous content verification are essential when considering ChatGPT’s clinical use. This is expected because ChatGPT was not designed for clinical applications.

Large language models such as clinicalBERT^24^ and BioGPT^25^, trained on domain-specific content, outperformed ChatGPT in clinical tasks^13^. These conflicting findings highlight the current lack of understanding regarding whether ChatGPT can effectively be used for clinical assessments. We next describe a mental health disorder for which clinical care can benefit by the use of ChatGPT analysis of text-based data.

Each year, approximately 140 million women give birth worldwide. For approximately one-third of this population, childbirth may be a source of substantial acute stress or trauma^26–29^, and a significant minority will develop childbirth-related post-traumatic stress disorder (CB-PTSD)^30,31^. Historically, PTSD has been associated with military combat or severe sexual assault^32^. In recent years, however, childbirth has become increasingly acknowledged as a significant PTSD trigger^30,33,34^.

Of the global childbearing population, approximately 6% will manifest full CB-PTSD^33^, which translates to 8 + million affected women per year. Untreated CB-PTSD is associated with negative effects in the mother and, by extension, her child^35,36^, and these consequences carry significant societal costs^37,38^. Early treatment for CB-PTSD facilitates improved outcomes^39^. This underscores the imperative need for effective strategies that can predict the development of CB-PTSD soon after a traumatic birth. Currently, the evaluation of CB-PTSD relies on clinician evaluations, which do not meet the need for a rapid, low-cost assessment. Patients’ self-reporting of their symptoms via questionnaires may entail under-reporting due to stigma, social desirability bias, fears of infant separation, and lack of awareness that can lead to significant under-diagnoses^40,41^.

Alternatively, the narrative style and language that individuals use when recounting traumatic events have been suggested to provide deep insights into their mental well-being^42–44^. Research has shown that the way in which individuals remember and describe traumatic events, encompassing the language used in the narrative, is connected to the expression of their post-traumatic stress symptoms^45^. The words in individuals’ trauma narratives may reflect post-trauma adjustment even before deep psychological analysis occurs^46^.

To date, the potential of using childbirth narratives analyzed via advanced text-based computational methods and ML for early detection of individuals showing signs of traumatic stress post-childbirth has been minimally explored (e.g.,^47^). We previously used the embeddings of sentence-transformers PLMs to train an ML classifier for identifying women at risk for developing CB-PTSD, using childbirth narratives as the data source; the model achieved good performance (F1 score of 0.76) in identifying women at risk of CB-PTSD via classification^47^. However, more research is required to characterize how word usage in birth narratives indicates maternal mental health, and understanding and analyzing trauma narratives remains a research area ripe for exploration.

This paper explores the capabilities of ChatGPT and the text-embedding-ada-002 (ADA) model, both developed by OpenAI, in analyzing childbirth narratives to identify potential markers of CB-PTSD. Through the lens of ChatGPT and associated models, we aim to bridge the gap between trauma narratives and early detection of psychiatric dysfunction, offering a novel approach to identifying women at risk for CB-PTSD. To achieve this aim, we collected textual narratives of recent childbirth experiences of postpartum women. Using OpenAI’s models, we tested whether the text of narratives, alone, could be used to identify postpartum women with probable CB-PTSD. To validate the developed model, we compare its performance to six previously published PLMs that were trained on medical or psychiatric domains.

## Materials and methods

### Study design

This investigation is part of a research study focused on the impact of childbirth experience on maternal mental health. Women who gave birth to a live baby in the last six months and were at least 18 years old participated by providing information about their mental health and childbirth experience through an anonymous web survey. Participants were given the opportunity to recount their childbirth stories at the end of the survey. These narratives were collected, on average, 2.73 ± 1.82 months post-childbirth (ranging from 0.02 to 8.34 months). The analyzed sample consists of 1295 women who provided narratives of length 30 words or more, which length was selected to facilitate meaningful analysis, consistent with previous work that noted limitations in analyzing shorter narratives^47,48^. Subject population characteristics are provided in Table 1.

**Table 1:** Subject population characteristics.

Variable	Value (%) or Mean (SD)
Maternal age (Years)	32.3 (4.4)
Education	
Formal college degree or higher	1067 (82.4%)
No formal degree	228 (17.6%)
Household income (US Dollars)	
< 20*,*000	34 (2.6%)
20*,*000–99*,*999	542 (42.1%)
100*,* 000–300*,*000	658 (51.2%)
> 300*,*000	52 (4.0%)
Marital status	
Married or domestic partnership	1214 (93.7%)
Single or divorced	81 (6.3%)
Primiparity	
Primiparas	689 (53.2%)
Multiparas	605 (46.8%)
Gestation week	38.9 (1.9)
Premature delivery	96 (7.4%)
Mode of delivery	
Vaginal	881 (68.0%)
Cesarean	414 (32.0%)
Obstetric complication in birth	433 (33.5%)
NICU admission	188 (14.6%)

N = 1295; Note that categories of variables with subsets that do not sum to 1295 are due to missing data. Cesarean: Planned, unplanned, emergency; NICU, Neonatal Intensive Care Unit; Premature Delivery: < 37 weeks of gestation; Vaginal: Natural, vaginal, and vaginal assisted.

Recruitment took place from November 2016 to April 2017, and from April 2020 to December 2020. Participants were recruited through hospital announcements, social media, and professional organizations. This study received exemption from the Partners Healthcare (Mass General Brigham) Human Research Committee (PHRC). All research was performed in accordance with the relevant guidelines and regulations. Implied consent was obtained from all participants; they were informed that by providing responses to the study measures, they are implying their consent to participate in the study. 

### Measures

We gathered narratives of childbirth in the form of open-ended, unstructured written text-based accounts, highlighting each participant’s personal and recent experience of childbirth. These narratives were procured using a free recall methodology, in which participants were asked to provide a brief account of their recent childbirth experience, focusing specifically on the most distressing elements, if any. This focus on the most distressing aspects of the birth experience aligns with standard procedures used in non-postpartum trauma sequelae research^49,50^.

For each participant, we assessed PTSD symptoms associated with childbirth using the Posttraumatic Stress Disorder Checklist for DSM-5 (PCL-5)^34,51^, a 20-item self-report measure employed to ascertain the presence and severity of DSM-5 PTSD symptoms after a designated traumatic event over the preceding month. The PCL-5 is widely recognized for its strong alignment with diagnostic assessments by clinicians and is used to establish a provisional PTSD diagnosis^34,52^ (i.e., a presumptive disease state without a formal diagnosis), and is validated in postpartum samples^34^. The clinical cutoff for this measure in non-postpartum samples is reported to be 31–33^53^, with a high specificity level using this cutoff^34^, and in accordance with this, and to reduce false negatives^34^, we used values of 31 + to define high scores for this study (potential CB-PTSD). The reliability of this tool was high, as indicated by Cronbach’s *α* = 0*.*934. For 14 participants, missing items in the PCL-5 assessment were coded as 0.

### Narrative analysis

**Figure 1:**
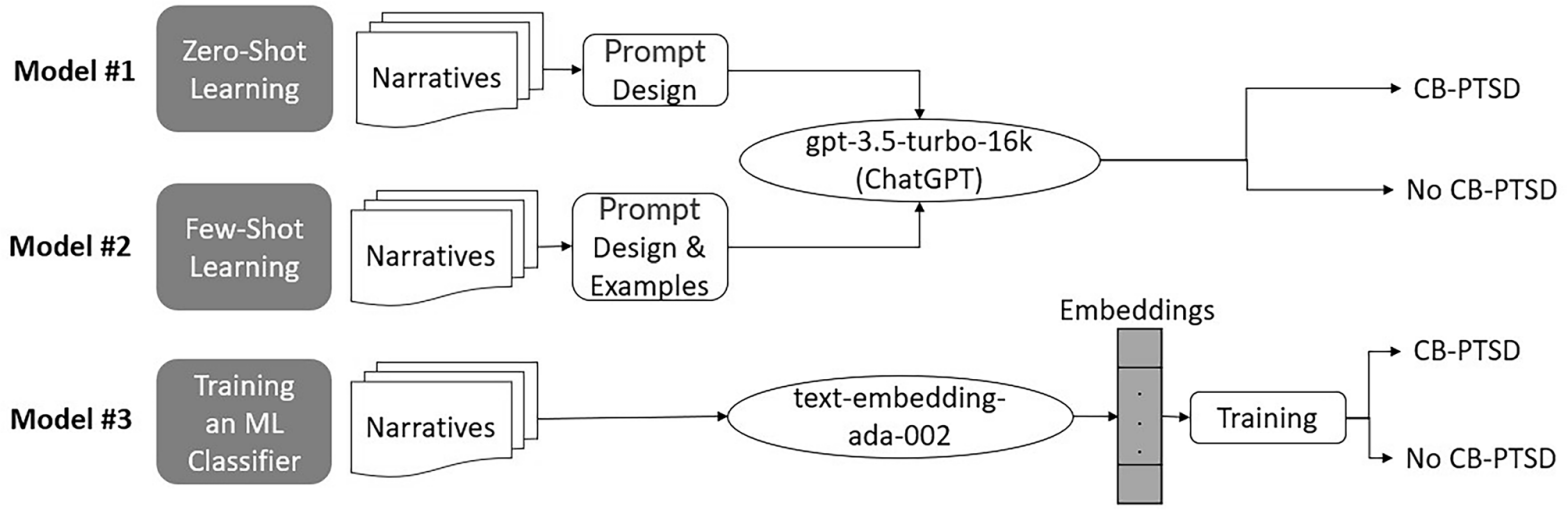
The three models employed in this study via OpenAI’s API: (1) Model #1 utilizes gpt3.5-turbo-16k for zero-shot classification, (2) Model #2 utilizes gpt3.5-turbo-16k for few-shot learning, and (3) Model #3 utilizes the text-embedding-ada-002 model to train a neural network machine learning (ML) model to screen via classification for childbirth-related post-traumatic stress disorder (CB-PTSD). In the Results section, we also present the results of testing Model #3 with other large text-embedding models.

We tested the performance of ChatGPT via two model configurations (Model #1: zero-shot classification, and Model #2: few-shot learning) by utilizing the gpt-3.5-turbo-16k Pre-trained Large Language Model (PLM) via OpenAI’s API. The gpt-3.5-turbo-16k PLM by OpenAI is a powerful transformer model that excels in natural language understanding and generation tasks. Its variation gpt-3.5-turbo-16k has the same capabilities as the standard gpt-3.5-turbo model but can process narratives that are four times longer, of up to 16,384 tokens. In addition, we tested the performance of our developed Model #3 that utilizes the embeddings of the text-embedding-ada-002 model via OpenAI’s API. Figure 1 presents a summary of the three tested models.

*Model #1—Zero-shot classification:* With no previous examples given to the model, we designed a prompt that includes a description of the task, followed by the narrative to be classified. The category associated with the model’s highest-confidence response was ‘1’ (Class 1: CB-PTSD) or ‘0’ (Class 0: No CB-PTSD) as the predicted class for the narrative. We experimented with several versions of prompts. As a summary, only the prompt that yielded the best results is presented (Table 2). Using OpenAI’s API, we sent this prompt to the gpt-3.5-turbo-16k model, which returned a response (1 or 0) to each prompt. The ‘temperature’ variable was set to 0, to make the model deterministic, i.e., always choosing the most likely next token in the sequence.

**Table 2:** Prompts for zero- and few-shot learning using ChatGPT.

Model	Prompt
Zero-shot	You are a psychiatrist specialized in diagnosing and treating Post-Traumatic Stress Disorder (PTSD). I will provide you with a narrative written by a woman describing her birth experience. Your task is to decide whether this woman is at high risk of PTSD (Label 1) or lower risk of PTSD (Label 0). Do not write anything but ‘1’ or ‘0’. ### < Text > : ”*{*text*}*”
Few-shot	You are a psychiatrist specialized in diagnosing and treating Post-Traumatic Stress Disorder (PTSD). I will provide you with a narrative written by a woman describing her birth experience. Your task is to decide whether this woman is at high risk of PTSD (Label 1), or lower risk of PTSD (Label 0). Do not write anything but ’1’ or ’0’. Here are a few examples of text with their associated class labels as ‘1’ (PTSD) or ‘0’ (No-PTSD). < Text > : “*{*PTSD narrative*}*” < Label > : 1 ### < Text > : “*{*No-PTSD narrative*}*” < Label > : 0 ### < Text > : “*{*text*}*”

*Model #2—Few-shot classification*:We provided two narratives (one written by a woman with CB-PTSD, and one written by a woman without CB-PTSD) and their associated labels in a conversation format to guide the model towards the classification task (Table 2). The gpt-3.5-turbo-16k model with ‘temperature’ = 0 then used these examples to classify the expected output for the subsequent narrative. Increasing the number of examples up to 4 provided similar model performances.

*Model #3—Training an ML classifier:* We converted narratives to numerical vector representations (embeddings) using the text-embedding-ada-002 model by OpenAI. The model takes narratives as input and generates 1536-dimensional embedding vectors, capturing relationships between words and phrases. These embeddings serve as inputs for our developed Model #3. More specifically, we trained a neural network (NN) ML model using the generated embeddings, to classify narratives as markers of endorsement (Class 1), or no endorsement (Class 0), of CB-PTSD. Appendix A presents the four steps to build and test Model #3, summarized in Fig. A1.

Note that all of the models tested in this study rely exclusively on textual features to identify CB-PTSD.

In Step #1 of Appendix A, we label narratives associated with PCL-5 ≥ 31 as ‘CB-PTSD’ (Class 1; 190 subjects), else ‘no CB-PTSD’ (Class 0; 1105 subjects).

In Step #2, we discarded narratives with < 30 words and balanced the dataset using down-sampling by randomly sampling the majority Class 0 to fit the size of the minority Class 1, resulting in 190 narratives in each class. We constructed the Train and Test datasets as described in Step #2, resulting in 170 narratives in each class.

To identify similar or contextually relevant narratives, which involve shared characteristics, content, and context, in Step #3 we adopted the approach used in our previous work^47^. This approach analyzes pairs of narratives as training examples, thus substantially increasing the number of training examples. We created three sets of sentence-pairs using the Train set: Set #1: All possible pairs of sentences (*C*(*n, r*) = *C*(1720*,* 2) = 14,365) in Class 1 (CB-PTSD). Set #2: All possible pairs of sentences (14,365) in Class 0. Set #3: Pairs of sentences (28,730), one randomly selected from Class 1 and another randomly selected from Class 0. We labeled sets #1 and #2 as positive examples as they contained semantically or contextually similar pairs of sentences (i.e., either a pair of narratives of individuals with, or without, CB-PTSD). We labeled set #3 as negative examples as it contained pairs of non-semantically or non-contextually similar pairs of sentences. This data augmentation process produced 57,460 training examples in the Train set.

Next, we mapped each narrative using the text-embedding-ada-002 model into a 1536-dimensional vector. Lastly, we computed the Hadamard product (*◦*)^54^ among each of the 57,460 embedding (emb) vectors of pairs of sentences (*u, v*) in sets #1 to #3 of the Train set (Appendix A), such that *z* = (*emb*(*u*) *◦ emb*(*b*)) (Appendix A).

Finally, using the 57,460 vectors, following the modeling approach in^47^, we trained a deep feedforward neural network (DFNN) model to classify pairs of sentences in sets #1 to #3 as semantically similar or not. DFNN models process information in one direction, and they enable the efficient processing of nonlinear data. Following preliminary work testing logistic regression and decision trees, which performed less accurately than DFNN, we elected to use a DFNN model. For training, we used the Keras Python library and constructed a DFNN with an input layer of 1536 neurons, 2 hidden layers of 400 and 50 neurons, and an output neuron. All layers had a rectified linear unit (ReLU) activation function, except the output neuron, which had a Sigmoid activation function. We used 50 epochs, applying the Adam optimizer with a learning rate of 1e^−4^, batch size of 32, and binary cross-entropy loss to monitor training performance. To avoid overfitting, we stopped training when there was no loss improvement for 3 consecutive epochs. We used 20% of the Train dataset for validation during the training process.

Steps #1 to #3 of Model #3 (Appendix A) were repeated 10 times to capture different narratives for creating an accurate representation of Classes 0 and 1.

*Model evaluation* In Step #4, Models #1 and #2 were evaluated on the entire dataset. Model #3 was trained on a Train set and evaluated on a Test set. This process was repeated 10 times, similar to a ten-fold cross-validation process. We tested and compared the performances of Models #1 to #3, using (1) the F1 score, which is a measure integrating precision (positive predictive value) and recall (sensitivity), and (2) the area under the curve (AUC).

Previous research reported that ChatGPT’s performance in the biomedical domain is moderate or satisfactory in various tests^13^. Currently, ChatGPT is not reliable for clinical deployment due to its design, which does not prioritize clinical applications^13^. Research indicates that specialized natural language processing (NLP) models trained on biomedical datasets remain the recommended approach for clinical uses^13^. Therefore, to evaluate our model, we compared the performance of our Model #3 with different embeddings generated by six PLMs that were trained on different domains, including clinical and mental health domains: all-mpnet-base-v2^47^, mental-roberta-base^6^, mental-bert-base-uncased^6^, mental-xlnet-base-cased^7^, Bio ClinicalBERT^24^, and BioGPT^25^. We used the HuggingFace repository^55^ with Python coding to work with the evaluated PLMs. The models that we compared were evaluated using two Evaluation Methods on the dataset published in^47^:

Evaluation Method 1: We fine-tuned each of the six evaluated PLMs on a down-stream task of classifying narratives as CB-PTSD (Class 1) or not (Class 0). We used 30% and 70% of the data for the Test and Train split, respectively.

Evaluation Method 2: We used the developed Model #3 with embeddings of the six evaluated PLMs. Following Step 2 of Appendix A, we split the Train and Test sets 10 times (similar to a ten-fold cross-validation process).

## Results

Following the data processing (Steps #1 and #2, Appendix A), for Class 1 (CB-PTSD) and Class 0 (no CB-PTSD), the mean word counts were 194.67 and 155.39, and median word counts were 158 and 106, respectively.

The results of applying Models #1 to #3 to the narrative datasets are presented in Table 3. Model #3 outperformed all other models in terms of AUC, F1 score, sensitivity.

**Table 3:** Comparison of models’ performance classification results. Model #1 was evaluated on the analyzed dataset. Model #2 was evaluated on the analyzed dataset, with the exception of two training examples that were used for few-shot learning. Model #3 was evaluated on the Test set of the analyzed dataset.

Model	AUC	F1 score	Sensitivity (Recall)	Specificity
Model #1	0.60	0.33	0.20	0.99
Model #2	0.60	0.38	0.24	0.96
Model #3	0.80	0.81	0.85	0.75

The results from ChatGPT Model #1 and Model #2 highlight a common challenge: these models struggle to classify narratives in a specific domain of expertise because they have not been trained on it. In other words, they are pre-trained models that have not been tailored to the specialized subject matter. Model #3, however, successfully addressed this problem and outperformed the other models. It did so by using 57,460 examples and being trained on the specific classification task. This specialized training used embeddings to create a classification system designed to detect CB-PTSD. By training the model in this way, it was better suited for the specialized task of CB-PTSD detection.

**Figure 2:**
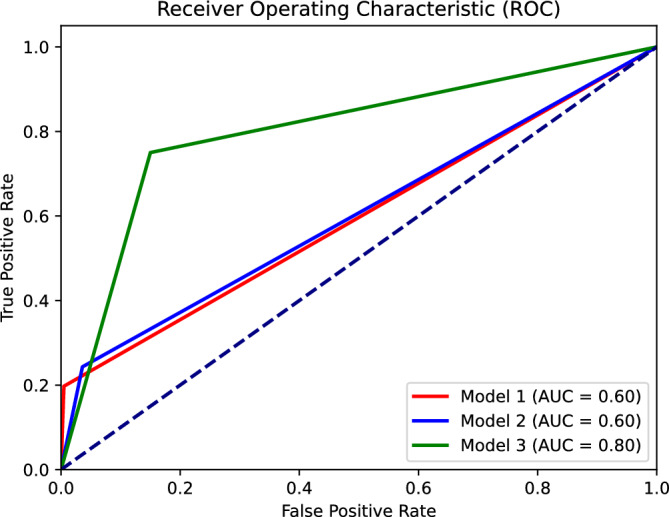
Comparison of Receiver Operating Characteristic (ROC) curves of binary classification Models #1 to #3. The three models directly predict class labels (0 or 1) instead of probabilities. The ‘stepped’ appearance of the ROC curves is due to the models’ binary output, which allows for only 0 or 1 thresholds.

As reported in Table 3 and, in particular, regarding the F1 score (0.81) and AUC (0.80), our model for CB-PTSD classification derived from birth narratives achieved overall good performance (Fig. 2).

Results of Evaluation Method 1: The results show F1 score lower than 0.2 for all PLM models.

Results of Evaluation Method 2: The results show (Table 4) that our Model #3 with OpenAI’s text-embedding-ada-002 embeddings outperformed Model #3 with other embeddings of PLMs (including embeddings of PLMs trained on clinical or mental health domains) in identifying CB-PTSD using narrative data only.

**Table 4:** Comparative performance analysis with different embeddings in Model #3 (Appendix A).

Model	AUC	F1 score	Sensitivity (Recall)	Specificity
Model #3	0.80	0.82	0.81	0.72
Model #3 with text-embeddings of All-mpnet-base-v2^47^	0.75	0.76	0.80	0.70
Model #3 with text-embeddings of Mental-roberta-base^6^	0.67	0.71	0.80	0.55
Model #3 with text-embeddings of Mental-xlnet-base-cased^7^	0.65	0.70	0.80	0.50
Model #3 with text-embeddings of Bio+ClinicalBERT^24^	0.65	0.63	0.60	0.70
Model #3 with text-embeddings of BioGPT^25^	0.62	0.59	0.55	0.70
Model #3 with text-embeddings of Mental-bert-base-uncased^6^	0.63	0.58	0.60	0.70

The average performance results of the ten-fold cross-validation process conducted on the same analyzed dataset that was used in^47^ are presented. Note: The dataset used here is a subset of the dataset used in Table 3. OpenAI’s text-embedding-ada-002 embeddings in Model #3 (first row in Table 4) outperform all other embeddings in Model #3, demonstrating superior ability in identifying CB-PTSD using narrative data only. Results are ordered by descending F1 score value.

## Discussion

AI- and ML-based analyses of free-text data sources via natural language processing (NLP), including the ChatGPT platform, hold significant promise for improving the assessment and diagnosis of mental health disorders. However, the examination of these technologies for mental health assessment remains in early stages, with previous findings about the utility of ChatGPT being mixed, and previous reports suggesting that ML models trained on application-specific corpuses of text might be necessary for accurate model performance. This study sought to explore the performance of different variations of ChatGPT, and text embedding of different models, for the purpose of identifying probable cases of childbirth-related post-traumatic stress disorder (CB-PTSD) using brief childbirth narratives of postpartum women.

The importance of prompt and accurate screening for CB-PTSD cannot be overstated^34,56^, as early interventions are essential to prevent the progression of this disorder to chronic stages, complicating treatment. Despite this pressing need, standardized CB-PTSD screening protocols are not yet established^57^. By assessing several model variations of ChatGPT, and narrative embeddings generated by different language models, we systematically studied the capabilities and shortcomings of these models to assess maternal mental health using childbirth narratives. While Model #1 (zero-shot learning) and Model #2 (few-shot learning) that utilize the pre-trained ChatGPT model exhibited limitations, Model #3, drawing from OpenAI’s text-embeddings-ada-002 embeddings, demonstrated superior performance in identifying CB-PTSD.

Notably, Model #3’s performance surpasses both the basic implementations of ChatGPT, and other PLMs trained in clinical and mental health domains, supporting its potential to offer richer insights into maternal mental health following traumatic childbirth. Our Model #3’s capability, assessed across the analyzed dataset, achieves 85% sensitivity and 75% specificity (Table 3) in identifying CB-PTSD cases based on narrative language. Additionally, Model #3 outperforms previously established models, such as the one presented in our recent previous work^47^.

In contrast, as reported in Table 3, ChatGPT, in its current iteration (gpt-3.5-turbo-16k), manifests only modest results, confirming its previously reported non-specialized nature for clinical applications^13,22,23^. Existing evaluations, including ours, frequently categorize ChatGPT as not suitable for healthcare data analysis, with its appropriate applications mostly limited to controlled research settings^13,22,23^.

Our unique approach, based on unstructured childbirth narratives, introduces an innovative, patient-friendly data acquisition method that may permit the early identification of women at risk of CB-PTSD before other strategies may detect symptoms of this condition. Additionally, women sharing narratives of their childbirth experiences may avoid problems associated with social desirability bias in questionnaire responses^41^, and may circumvent under-reporting of symptoms due to shame or fear^58^. Preliminary assessments based on these narratives can identify high-risk women, facilitating timely medical intervention. Our model’s exclusive reliance on childbirth narratives as its data source presents an efficient mechanism for data collection during the vulnerable postpartum stage, circumventing potential pitfalls of using only medical records. The proposed model has the potential to fit seamlessly into routine obstetric care, and may serve as a foundation for commercial product development, facilitating its mainstream adoption. Importantly, this could improve the accessibility of CB-PTSD diagnosis, addressing socioeconomic, racial, and ethnic disparities associated with childbirth trauma^59,60^ by helping to identify minoritized women, who are at three times higher risk of experiencing post-traumatic stress symptoms^60^.

A period of six months postpartum involves the extended postpartum period, which is an important time for the establishment of chronic PTSD symptoms. However, the childbirth narratives analyzed in our study were collected, on average, 2.73 ± 1.82 months post-childbirth (range: 0.02 to 8.34 months). This indicates that our model can be used for early classification of women with and without CB-PTSD to offer an early tool for accurate identification and therefore support the possibility for early treatment.

While our results are promising, our study has several limitations. The potential enhancement of our model with data from additional sources remains unexplored; these sources can include patient self-report questionnaires, medical record data that might indicate the presence of birth trauma, and physiological assessments (e.g.,^61^). The sample includes women who gave birth before and during the COVID-19 pandemic^62,63^. This heterogeneity, including the possibility that women used different language in their narratives during the pandemic, may have affected our results and warrants replication in other postpartum samples. Additionally, while we assessed the presence of CB-PTSD using the PCL-5, which is a well validated self-report measure ^34,51,64,65^ and shows strong correspondence with clinical diagnostics^34^, clinician evaluations were not performed. A more diverse subject population is needed in future work to facilitate the development of a universally applicable tool for CB-PTSD assessment. Moreover, any use of PLM technology for mental health research warrants considerations involving the reliability of the content provided by ChatGPT and other PLMs^66,67^, and security and privacy concerns involving PLM analysis of medical texts^66,67^. Additionally, external validation is essential to further corroborate our findings using the DFNN model that employs text embeddings (Model #3).

Looking forward, we advocate for two principal enhancements to our model to identify CB-PTSD in postpartum women based on their childbirth narratives: (i) Specific fine-tuning of ChatGPT for CB-PTSD narrative language, optimizing embedding vector representation; and (ii) The integration of additional data types, including electronic medical records. Such augmentations can serve to enhance performance, improving the accuracy of computational methodologies in maternal mental health evaluation.

## Conclusions

In this investigation, we examine the utility of variations of the ChatGPT pre-trained large language model (PLM), and text embeddings of different language models to assess mental health using text-based personal narratives as the exclusive data source. Harnessing advanced natural language processing (NLP) and machine learning (ML) analysis strategies, we present the potential of these methods for analyzing narratives to advance maternal mental health assessment. We find that a ChatGPT model untrained on a specific clinical task shows inadequate performance in the task of identifying childbirth-related post-traumatic stress disorder (CB-PTSD), while our model trained on the embeddings of OpenAI’s text-embeddings-ada-002 model yields the best performance to date for this task, representing good performance. With refinements and enhancements pending in future work, this textual personal narrative-based assessment strategy employing NLP analysis has the potential to become an accurate, efficient, low-cost, and patient-friendly strategy for identifying CB-PTSD in the clinic, and facilitating timely interventions to mitigate this maternal mental health disorder. The PLM analysis strategies presented here hold promise for potential use in assessing diverse additional mental health disorders, and consequently improving outcomes.

### Supplementary information

Supplementary Information 1.

## Data Availability

The de-identified datasets used and analyzed in the current study are available from the corresponding author on reasonable request.

## Code availability

The code associated with this study is publicly available on GitHub at https://github.com/bartala/ChatCBPTSD.
