# Supplementary material for: AI and narrative embeddings detect PTSD following childbirth via birth stories
Source: Sci Rep. 2024 Apr 11;14:8336. doi: 10.1038/s41598-024-54242-2 (PMC11009279; doi:10.1038/s41598-024-54242-2)
Supplement: Supplementary file 1 — Supplementary Information 1. [file 41598_2024_54242_MOESM1_ESM.pdf]

# Appendix A:
# AI Trained on Text Embeddings of Birth Narratives Detects Post-Traumatic Stress Disorder Following Childbirth

Alon Bartal[1], Kathleen M. Jagodnik[1,2,3], Sabrina J. Chan[2], Sharon Dekel[2,3*]

[1]The School of Business Administration, Bar-Ilan University, Ramat Gan, 5290002, Israel.
[2]Department of Psychiatry, Massachusetts General Hospital, Boston, 02114, Massachusetts, USA.
[3]Department of Psychiatry, Harvard Medical School, Boston, 02115, Massachusetts, USA.

*Corresponding author. E-mail: sdekel@mgh.harvard.edu
Contributing authors. Emails: alon.bartal@biu.ac.il; kathleen.jagodnik@biu.ac.il; sabrina_chan@dfci.harvard.edu

## Appendix A Steps to Build and Test Model #3 of This Study

The following four steps describe how we built and tested Model #3:

**Step 1. Define a PCL-5 cutoff score.** We labeled each narrative as Class 1: Probable CB-PTSD ('CB-PTSD') based on PCL-5 ≥ 31, or Class 0: No Probable CB-PTSD ('No CB-PTSD') based on PCL-5 < 31.

**Step 2. Data preparation.** We discarded narratives with < 30 words from the dataset [47,48]. To handle the imbalance in the analyzed dataset (due to the small representation of cases with PCL-5 ≥ 31), we randomly sampled the majority Class 0 to fit the size of the minority Class 1. Using the balanced dataset, we randomly selected 70% of the narratives to train our model and 30% to test our model. This step was repeated 10 times.

**Step 3. Develop a Machine Learning (ML) classifier that utilizes Natural Language Processing (NLP) features.** Using the Train set, we develop a model that analyzes pairwise narrative (sentence) data. The goal of the developed model is to learn how to identify semantically or contextually similar pairs of sentences. First, each sentence ($S_i$) is mapped onto a fixed-size embedding vector using the text-embedding-ada-002 model via OpenAI API, which we denote $emb(S_i)$. Thus, $S_i$ is encoded to a vector using a function $emb(S_i)$. To learn if two sentences are semantically or contextually similar, we train a classifier to analyze their Hadamard product (denoted: $\circ$ ) [54] and decide whether they affiliate with the same class or not. Given a sentence $S_a$ not present during training, a sentence $S_1 \in$ Class 1, and a sentence $S_0 \in$ Class 0, then $S_a \in$ Class 1 if the probability of Class 1 affiliation when applying our developed model to the Hadamard product $emb(S_a) \circ emb(S_1)$ is higher than the probability of Class 0 affiliation when applying our developed model to $emb(S_a) \circ emb(S_0)$.

This approach allowed us to generate multiple training examples since there are $n(n-1)/2$ possible combinations for $n$ sentences, thus addressing the challenge of training an ML model with a low number of examples, as in Class 1. More specifically, the following three substeps describe the model development.

1. Each pair of sentences in Class 1, and each pair of sentences in Class 0, were labeled as positive examples, indicating semantically or contextually similar sentences of individuals with (Set #1) or without (Set #2) CB-PTSD, respectively. Next, negative examples (Set #3) of the same size as the positive examples sets (||Set #1|| + ||Set #2||) were created by randomly selecting pairs of sentences, one from Class 1, and the other from Class 0, indicating semantically or contextually nonsimilar sentences.
2. Using the text-embedding-ada-002 model, each sentence was mapped into a dense vector space. Then, for each Set #1 to #3, we computed a vector $z$ of the embedding ($emb$) of each pair of sentences $(u, v)$, selected in

Substep 1, $z = (emb(u) \circ emb(v))$.

3. A densely connected feedforward neural network (DFNN) was trained to classify pairs of sentences (by processing vector $z$) as semantically similar or not.

**Step 4. Test model performance.** We compared the performance of Model #3 with the model in [47], as well as with Model #1 and Model #2. We report the area under the receiver operating characteristic curve (AUC), F1 score, Sensitivity, and Specificity measures on the Test set. To test Model #3 on a newly unseen narrative $S$ in the Test set, we first compute its embeddings. Next, we calculate the average embedding vector ($v_n$) of all Train narratives in Class 0, and the average embedding vector ($v_p$) of all Train narratives in Class 1. To decide the class of S, we compute $z_n = (emb(S) \circ v_n)$, and $z_p = (emb(S) \circ v_p)$. Then, we apply Model #3 (denoted as $f(x)$) to $z_n$ and $z_p$, and compare its output, i.e., compare the likelihood of similarity of $emb(S)$ to $v_p$ with the likelihood of similarity of $emb(S)$ to $v_n$. If $f(z_p) > f(z_n)$, we say that $S \in$ Class 1, else $S \in$ Class 0. Intuitively, our model should assign a higher likelihood of similarity between an embedded narrative of a woman with CB-PTSD to the vector $v_p$ than to the vector $v_n$.

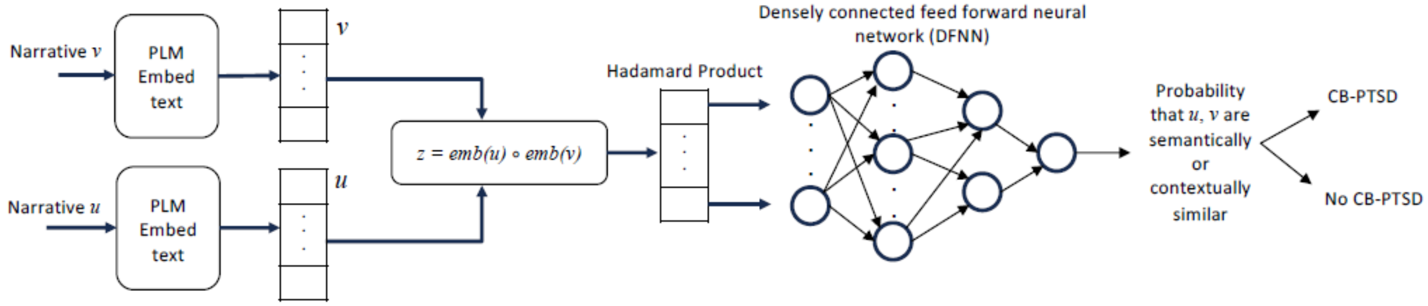


**Figure A1.** The modeling approach for classifying pairs of narratives associated with women with or without CB-PTSD.
